# Urine and Serum Metabolite Profiling of Rats Fed a High-Fat Diet and the Anti-Obesity Effects of Caffeine Consumption

**DOI:** 10.3390/molecules20023107

**Published:** 2015-02-13

**Authors:** Hyang Yeon Kim, Mee Youn Lee, Hye Min Park, Yoo Kyoung Park, Jong Cheol Shon, Kwang-Hyeon Liu, Choong Hwan Lee

**Affiliations:** 1 Department of Bioscience and Biotechnology, Kon-Kuk University, Seoul 143-701, Korea; E-Mails: festivalkim@naver.com (H.Y.K.); kkamlice@hanmail.net (M.Y.L.); ramgee@naver.com (H.M.P.); 2 Department of Medical Nutrition, Graduate School of East-West Medical Science, Kyung Hee University, Gyeonggi-do 446-791, Korea; E-Mail: ypark@khu.ac.kr; 3 College of Pharmacy and Research Institute of Pharmaceutical Sciences, Kyungpook National University, Daegu 702-701, Korea; E-Mails: sleier7640@naver.com (J.C.S.); dstlkh@gmail.com (K.-H.L.)

**Keywords:** obesity, caffeine, high-fat diet, mass spectrometry, multivariate analysis

## Abstract

In this study, we investigated the clinical changes induced by a high fat diet (HFD) and caffeine consumption in a rat model. The mean body weight of the HFD with caffeine (HFDC)-fed rat was decreased compared to that of the HFD-fed rat without caffeine. The levels of cholesterol, triglycerides (TGs), and free fatty acid, as well as the size of adipose tissue altered by HFD, were improved by caffeine consumption. To investigate the metabolites that affected the change of the clinical factors, the urine and serum of rats fed a normal diet (ND), HFD, and HFDC were analyzed using ultra performance liquid chromatography quadruple time-of-flight mass spectrometry (UPLC-Q-TOF-MS), gas chromatography (GC-TOF-MS), and linear trap quadruple mass spectrometry (LTQ-XL-MS) combined with multivariate analysis. A total of 68 and 52 metabolites were found to be different in urine and serum, respectively. After being fed caffeine, some glucuronide-conjugated compounds, lysoPCs, CEs, DGs, TGs, taurine, and hippuric acid were altered compared to the HFD group. In this study, caffeine might potentially inhibit HFD-induced obesity and we suggest possible biomarker candidates using MS-based metabolite profiling.

## 1. Introduction

Obesity is a chronic disease caused partially by the dietary habit of consuming excessive nutrients, especially those with high-fat content. In 2008, the U.S. Centers for Disease Control and Prevention (CDC) reported that 33.8% of American adults were considered obese. High-fat diet (HFD)-related obesity causes many complications, including type 2 diabetes, dyslipidemia, and cardiovascular diseases such as hypertension and atherosclerosis [1]. Obesity-related healthcare costs are $100 billion annually, and obesity itself results in the loss of societal and economic productivity [2]. In Korea, the prevalence of people who were obese and overweight in 2010 was 8.6% and 7.8%, respectively [3]. Although this is lower than obesity rates reported for the USA, the rate of obesity has increased gradually over 10 years (1997–2007) [4]. HFD-related obesity has been reported by many studies to be involved in the regulation of gut microbiota, cholesterol circulation, liver damage [5,6], and the mechanism of resistance to diet-induced obesity in germ free mice [7]. Recently, mass spectrometry (MS)-based metabolomics approaches, including metabolite profiling, metabolic fingerprinting, and target analysis, have been used to identify metabolites altered by a HFD [8,9].

Metabolomics identifies the different metabolites of target subjects or groups. It is a useful tool in diagnosing obesity [8] and obesity-related diseases such as type 2 diabetes [10], cardiovascular disease [11], and cancer [12,13]. Metabolomics techniques have also been used to examine the effect of an anticancer agent [14] and to monitor drug metabolism during new drug discovery [15]. However, metabolite profiling for the identification and elucidation of the biomarkers of consumption of an antiobesity substance such as caffeine has not been well reported. 

Caffeine is the major alkaloid ingredient of the coffee plant, tea bush, and cocoa [16] and can be found in colas, ice tea, and a number of energy drinks [17]. Moderate consumption of caffeine is helpful in reducing the risk of type 2 diabetes, hepatic disease [18], and cardiovascular disease caused by HFD [19], since caffeine inhibits fat accumulation and stimulates lipid metabolism in the liver [20,21]. However, the relationship between caffeine and obesity has not been fully elucidated. Therefore, to investigate the effects of caffeine on obesity, we profiled the metabolites in the urine and serum from rats fed a HFD and HFD with caffeine (HFDC) using comprehensive MS techniques such as ultra-performance liquid chromatography quadruple time-of-flight mass spectrometry (UPLC-Q-TOF-MS), gas chromatography (GC-TOF-MS), and linear trap quadruple mass spectrometry (LTQ-XL-MS) with multivariate analysis.

## 2. Results and Discussion

### 2.1. Clinical Data

In this study, we investigated the clinical changes induced by an HFD or HFDC in the serum, liver, and adipose tissue of rats compared with those on a normal diet (ND). After 12 weeks of HFD, the mean body weight of the HFD group was higher than that of the ND group (Table 1). After 9 more weeks, the triglyceride (TG) level in the serum and liver of the HFD group increased 1.39- and 5.75-fold, respectively, compared with the ND group. In addition, the total lipid in the liver accumulated to a level, which was 4.45-fold greater in the HFD group than the ND group. In the HFD group, higher levels of total cholesterol (TC) and low-density lipoprotein cholesterol (LDL-C) in the liver and serum were detected than the ND group. 

**Table 1:** Metabolic parameters and clinical data of rat serum, liver, and adipose tissue of different treatment groups.

Outcome Variables	ND ^(1)^			HFD ^(2)^			HFDC ^(3)^		
Body weight (g) ^(4)^									
After 12 weeks	502.63	±	20.43 ^a^	628.55	±	46.22 ^b^	628.79	±	46.36 ^b^
After 21 weeks	573.95	±	42.68 ^a^	747.74	±	69.70 ^c^	636.30	±	51.58 ^b^
Serum (mg/dL)									
Total cholesterol	92.21	±	20.25 ^b^	131.77	±	40.31 ^a^	89.39	±	23.21 ^b^
HDL-cholesterol	58.12	±	23,44 ^b^	33.25	±	12.59 ^a^	40.58	±	11.17 ^ab^
LDL-cholesterol	30.66	±	15.70 ^b^	63.03	±	22.96 ^a^	35.12	±	15.86 ^ab^
Triglyceride	83.42	±	20.64 ^b^	115.99	±	22.01 ^a^	83.99	±	24.61 ^b^
Free fatty acid	36.90	±	5.00 ^a^	26.19	±	4.96 ^b^	35.06	±	6.95 ^a^
Liver (μg/mg)									
Total cholesterol	2.90	±	0.65 ^b^	4.92	±	1.08 ^a^	2.78	±	1.27 ^b^
Triglyceride	13.61	±	5.71 ^b^	78.28	±	20.92 ^a^	17.71	±	13.93 ^b^
Total lipid	23.2	±	5.48 ^c^	103.29	±	9.52 ^a^	61.68	±	6.16 ^b^
Adipose tissue									
Adipocytes area (μm^2^)	632.36	±	157.07 ^ab^	747.12	±	154.71 ^a^	534.04	±	117.72 ^b^
Cell number per spot	147.57	±	30.61 ^a^	106.38	±	21.27 ^b^	151.22	±	31.61 ^a^
Adipose tissue enzyme activities									
GPDH (nmol/min/mg)	22.61	±	5.29 ^a^	43.40	±	22.67 ^b^	24.76	±	14.16 ^ab^
LPL (nmol/min/mg)	21.75	±	13.23 ^a^	71.00	±	19.75 ^c^	27.82	±	15.74 ^a^

^(1)^ ND (normal diet): Normal diet (Harlon 2018S) + Distilled water (*n* = 10); ^(2)^ HFD (high fat diet): 60 kcal% fat + Distilled water (*n* = 9); ^(3)^ HFDC (high fat diet with caffeine): 60 kcal% fat + 0.1% Caffeine solution (*n* = 10); ^(4)^ After 12 weeks of only a HFD or ND, or after 21 weeks after drinking caffeine for 9 weeks (HFDC group). Data are expressed as mean ± SD. Statistical difference between experimental groups is based on analysis using one-way ANOVA and Tukey’s multiple range test at *p* < 0.05. Means with different letters (e.g., a or b) are statistically different. Differences were considered significant at *p* < 0.05.

In addition, the size of the adipose tissue and activities of glycerol-3-phosphate dehydrogenase (GPDH) and lipoprotein lipase (LPL) were greater in the HFD group than in the ND group. In contrast, the high-density lipoprotein cholesterol (HDL-C) and free fatty acid concentrations in the serum were lower in the HFD group than the ND group. These changes in the clinical data of the HFD group were observed in many studies that compared values between a normal and overweight/obese group [8,22,23,24]. Providing a HFD for 21 weeks resulted in rats that were overweight/obese, as predicted by the model. After 9 weeks of caffeine consumption, the mean body weight of the HFDC group was maintained, while the weight of the rats fed a HFD and ND gradually increased (Figure S1). At the end of the study, the mean body weight and adipose tissue size of the HFDC group were significantly lower than the HFD group. Moreover, lipid levels such as TC and TG in the serum and liver were decreased in the HFDC group, accompanied by decreased GPDH and LPL enzyme activities, both of which are indicators of lipid metabolism and function to increase TG accumulation [25,26]. A number of studies have demonstrated that weight gain caused by increasing fat content is delayed by caffeine consumption in the HFD group [20,27]. In addition, Sinha *et al.* [28] have reported that caffeine induces autophagy and increases lipolysis in the hepatic cell, which plays a negative role in fat accumulation. Thus, our results demonstrate that caffeine consumption affected the lipid metabolism in rats fed a HFD. 

### 2.2. Analysis of Urine Metabolite Profiling 

**Figure 1:**
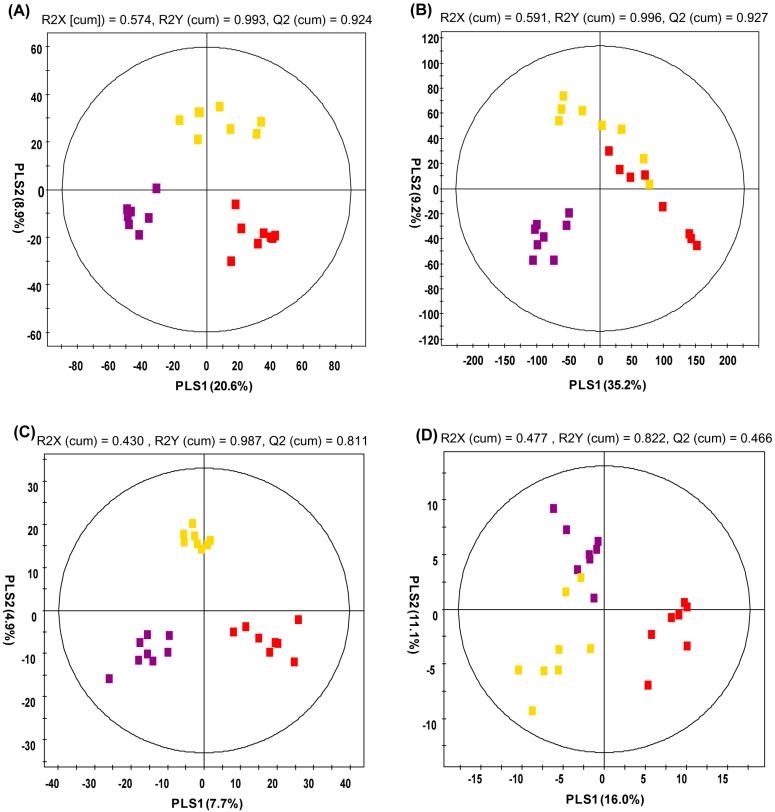
Partial least-squares discriminant analysis (PLS-DA) score plots. Urine analysis by (**A**) UPLC-Q-TOF-MS and (**B**) GC-TOF-MS. Serum analysis by (**C**) UPLC-Q-TOF-MS and (**D**) LTQ-XL-MS. (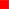: normal diet; 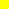: high-fat diet, 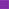: high fat diet with caffeine).

Urine metabolite profiling was analyzed using UPLC-Q-TOF-MS and GC-TOF-MS. In the partial least-squares discriminant analysis (PLS-DA) score plot by UPLC-Q-TOF-MS (Figure 1A), the ND and HFDC groups were clearly differentiated by PLS component 1, while the HFD group was discriminated from the ND and HFDC groups by PLS component 2. The urine PLS-DA score plot for GC-TOF-MS (Figure 1B) was similar to that of the UPLC-Q-TOF-MS. The metabolites selected by variable importance in projection (VIP) values (>0.7) in the UPLC-Q-TOF-MS and GC-TOF-MS are presented in Table 2 and Table 3, respectively. Higher VIP value represents a higher correlation between metabolites and groups. Twenty-eight and forty urine metabolites were selected as significant variables using the UPLC-Q-TOF-MS and GC-TOF-MS, respectively. These variables were putatively identified by matching accurate mass, i-FIT value, relative retention time, and formula, with references, commercial library, standard compounds, and the human metabolome database (HMDB). Five metabolites were tentatively identified as hydroxyadipic acid, dihydroferulic acid 4-*O*-glucuronide, 3-indole carboxylic acid glucuronide, 5-hydroxy-6-methoxy-indole glucuronide, and 3-methyldioxyindole. Eight additional metabolites were not identified by the UPLC-Q-TOF-MS. In addition, 15 metabolites were only detected in HFDC group, which seemed to be caffeine metabolites. Of the five tentatively identified metabolites, the hydroxyadipic acid level was increased in the HFD group compared with the ND group, while the levels of four other metabolites including glucuronide-conjugated compounds and 3-methyldioxyindole were decreased. Furthermore, the level of two glucuronide-conjugated compounds in the HFDC group—dihydroferulic acid 4-*O*-glucuronide, and 3-indole carboxylic acid glucuronide—were increased by caffeine consumption compared with HFD group, but were not significant. Glucuronide-conjugated compounds are generated by UDP-glucuronosyltransferase (UGTs) enzymes in the liver of humans and rats in order to increase the solubility of toxins or xenobiotics for excretion [29]. HFD-induced obesity results in the expression of inflammatory markers, and it is related to the dysregulation of UGT levels in the fatty liver [30,31,32,33]. Moreover, Congiu *et al.* [34] have reported that patients with a high inflammation score have reduced UGT mRNA levels in the liver. Previous research has also demonstrated that caffeine treatment in obese rats for 9 weeks ameliorates pro-inflammatory cytokine production [35]. Thus, we inferred that the decreased levels of the glucuronide-conjugated metabolites might be due to the reduction of the gene expression of UGTs associated with HFD-induced inflammation and that caffeine consumption perhaps helps alleviate the level of glucuronide-conjugated metabolites induced by HFD. For these reasons, two glucuronide-conjugated compounds, dihydroferulic acid 4-*O*-glucuronide and 3-indole carboxylic acid glucuronide, might be potential biomarker candidates of HFD and caffeine consumption. However, the relationship between urinary glucuronide-conjugated compounds and HFD consumption has not yet been properly examined.

Of the variables identified by GC-TOF-MS (Table 3), the levels of most of the compounds, except hippuric acid, were increased in the HFD group compared with the ND group. The levels of monosaccharides such as xylose, ribose, and mannose, as well as sugar alcohols such as xylitol, adonitol, and mannitol, were presented as significantly different metabolites between the ND and HFD groups. Because glycolysis is suppressed and gluconeogenesis is upregulated in the HFD group [36], monosaccharides were anticipated to increase in the urine of the HFD group. In previous findings, the alteration of pseudouridine, creatinine, taurine, and hippuric acid levels in HFD group showed similar patterns when compared to our results [36,37]. According to one study, HFD intake was related to an increase in lipid oxidation, lipid accumulation, and changes in choline, purine, and amino acid metabolism. Another study reported that these metabolic changes in obesity positively correlate with chronic low-grade inflammation in various organs [38]. Moreover, the elevated levels of taurine and pseudouridine were interpreted as indicators of liver damage induced by inflammation related to lipid β-oxidation and oxidative stress [36,39,40]. Many researchers also suggest that the alteration of gut microbiota by HFD exacerbates inflammation and obesity [30,32]. Hippuric acid is derived from phenylalanine, and its metabolite is represented in a lower proportion in the urine of the HFD group due to the modulation of the gut microbiota population [37]. The results of the comparison of GC-TOF-MS results in the ND and HFD group suggested that the alteration of these metabolites was associated with overall metabolic dysfunction. Of the metabolites, the levels of taurine and hippuric acid were decreased and increased in the HFDC group, respectively, but were increased and decreased in the HFD group, respectively. Although those levels were not comparable to the ND group, caffeine consumption narrowed the gap between the ND and HFDC groups, compared with the ND and HFD. Therefore, the taurine and hippuric acid could be suggested as biomarker candidates of caffeine consumption in HFD-induced obesity. 

By investigating urine in the ND, HFD, and HFDC group using UPLC-Q-TOF-MS and GC-TOF-MS analysis, biomarker candidates such as glucuronide-conjugated compounds, taurine, and hippuric acid were suggested. These molecules were affected by the HFD or caffeine consumption, and indirectly related to obesity and overall metabolic dysfunction.

### 2.3. Analysis of Serum Metabolite Profiling 

Serum metabolites from the rats were analyzed using UPLC-Q-TOF-MS and LTQ-XL-MS. Both PLS-DA score plots were divided into three groups based on the PLS1 and PLS2 components (Figure 1C,D). 

A total of 10 and 42 significantly different metabolites in the three groups were selected by UPLC-Q-TOF-MS and LTQ-XL-MS analysis, respectively. All putatively identified metabolites, including bile acids, lysophosphatidylcholines (lysoPCs), phosphatidylcholines (PCs), diacylglycerols (DGs), cholesteryl esters (CEs), and TGs, were associated with lipid metabolism (Table 4 and Table 5). In the HFD group, the levels of lysoPC 14:0, 18:0, and 20:3, and lysoPC-P 18:0 were increased, while the lysoPC 16:1, 18:2, and 20:5, and PC 34:2 and 34:0 levels were decreased compared to the ND group. LysoPC is converted from PC by phospholipase A2, which is activated under inflammatory conditions such as with a HFD [41,42]. Many studies showed lysoPCs as significant biomarkers of mice fed a HFD [8], overweight/obesity humans [23], and obesity-resistant BALB/c mice [43]. The level of each specific lysoPC and PC was different, and it is coincident with our results.

**Table 2:** Discriminated metabolites of urine identified using UPLC-Q-TOF-MS.

RT (min)	Measured MS (*m/z*)		Tentative Metabolites	Molecular Formula		Error (mDa)		Adduct	Fold Change ^(1)^		
	Positive	Negative							HFD/ND	HFDC/HFD	HFDC/ND
3.01	180.0844	-	Hydroxyadipic acid	C_6_H_10_O_5_		−2.8		[M+NH_4_]^+^	1.682 *	0.875	1.472
3.47	395.0942	393.0828	Dihydroferulic acid 4-*O*-glucuronide	C_16_H_20_O_10_		−3.0		[M+H]^+^	0.018 *	9.641	0.176 *
3.69	338.0864	336.0749	3-Indole carboxylic acid glucuronide	C_15_H_15_NO_8_		−5.3		[M+H]^+^	0.071 *	3.864	0.273
3.93	340.0992	338.0895	5-hydroxy-6-methoxyindole glucuronide	C_15_H_17_NO_8_		−4.0		[M+H]^+^	0.024 *	0.066	0.002 *
4.38	164.0714	162.0549	3-Methyldioxyindole	C_9_H_9_NO_2_		0.2		[M+H]^+^	0.003 *	6.066	0.016
Caffeine metabolites ^(2)^													
1.50	171.0899	-	5-Acetylamino-6-amino-3-methyluracil (AAMU)	C_7_H_10_N_4_O_3_		1.7		[M+H−CO]^+^			
2.57	167.0570	-	7-Methylxanthine	C_6_H_6_N_4_O_2_		0.1		[M+H]^+^			
2.61	183.0529	-	1-Methyluric acid	C_6_H_6_N_4_O_3_		1.1		[M+H]^+^			
2.71	167.0569	-	3-Methylxanthine	C_6_H_6_N_4_O_2_		0.0		[M+H]^+^			
2.77	197.0675	-	3,7-Dimethyluric acid	C_7_H_8_N_4_O_3_		0.0		[M+H]^+^			
2.79	167.0567	-	1-Mehtylxanthine	C_6_H_6_N_4_O_2_		−0.2		[M+H]^+^			
2.97	197.0679	-	1,3-Dimethyluric acid	C_7_H_8_N_4_O_3_		0.4		[M+H]^+^			
3.03	181.0731	-	Theobromine	C_7_H_8_N_4_O_2_		0.5		[M+H]^+^			
3.20	197.0659	-	1,7-Dimethyluric acid	C_7_H_8_N_4_O_3_		−1.6		[M+H]^+^			
3.30	181.0733	-	Paraxanthine	C_7_H_8_N_4_O_2_		0.7		[M+H]^+^			
3.40	211.0835	-	1,3,7-Trimethyluric acid	C_8_H_10_N_4_O_3_		0.4		[M+H]^+^			
3.69	195.0888	-	Caffeine	C_8_H_10_N_4_O_2_		0.6		[M+H]^+^			
Not-identified													
0.87	109.0762	-	N.I.	-		-		-	2.111	0.291 *	0.614
1.41	126.0916	-	N.I.	-		-		-	1.825 *	1.955 *	3.568 *
1.88	127.0480	-	N.I. ^(2)^	-		-		-			
2.90	185.1050	-	N.I. ^(2)^	-		-		-			
3.09	185.1057	-	N.I. ^(2)^	-		-		-			
3.68	447.2227	-	N.I.	-		-		-	5.643 *	0.738	4.163 *
3.69	393.1527	-	N.I.	-		-		-	1.972 *	1.127	2.222 *
4.80	170.0599	-	N.I.	-		-		-	0.245 *	1.27	0.312 *
5.82	231.1425	-	N.I.	-		-		-	3.263 *	0.771	2.514 *
6.15	354.1368	-	N.I.	-		-		-	0.439 *	1.097	0.481 *
9.47	415.3032	-	N.I.	-		-		-	1.286	0.689 *	0.887

^(1)^ Fold change was calculated by dividing the mean of the peak intensity of each metabolite from each of the two groups; ^(2)^ Detected only in the high fat diet with caffeine (HFDC) group; RT, Retention time; N.I., Not identified; ND, normal diet; HFD: high fat diet; HFDC, high fat diet with caffeine; * *p* value < 0.05, - not detected.

**Table 3:** Discriminated metabolites of the urine identified using GC-TOF-MS.

RT (min)	MS Fragment	Tentative Metabolites	Derivatized	Fold Change ^(1)^			Ref. ^(2)^
				HFD/ND	HFDC/HFD	HFDC/ND	
5.21	45, 73, 116, 147	L-Alanine	(TMS)_2_	1.932 *	1.321	2.552 *	STD
7.56	45, 59, 73, 75, 100, 102, 117, 133, 147, 189, 292	Propanoic acid	(TMS)_3_	1.675	2.110 *	3.536 *	Lib
7.83	45, 73, 100, 147, 204, 218	L-Serine	(TMS)_3_	2.175 *	1.259	2.737 *	STD
8.08	45, 57, 73, 101, 117, 147, 219, 291	L-Threonine	(TMS)_3_	2.622 *	1.226	3.215 *	STD
8.14	45, 59, 73, 147, 255	Ethanesulfonic acid	(TMS)_3_	15.649 *	0.817	12.780 *	Lib
8.51	45, 53, 73, 75, 82, 109, 147	3-Methyl glutaconic acid	(TMS)_2_	2.323 *	1.050	2.440 *	Lib
9.14	45, 73, 103, 117, 147, 217	L-Threitol	(TMS)_4_	2.143 *	1.267	2.715 *	Lib
9.44	45, 73, 117, 147, 220, 292	L-Threonic acid	(TMS)_4_	2.121 *	1.222	2.592 *	Lib
9.54	45, 73, 115, 143, 171, 329	Creatinine enol	(TMS)_3_	2.762 *	1.111	3.070 *	Lib
10.40	45, 73, 103, 147, 217, 307	Xylose	(TMS)_5_	2.149 *	1.460	3.139 *	STD
10.44	45, 59, 73, 100, 147, 326	Taurine	(TMS)_3_	1.952 *	0.787	1.536 *	STD
10.51	45, 73, 103, 147, 217	Ribose	(TMS)_5_	2.914 *	0.722	2.105 *	STD
10.82	45, 73, 103, 129, 147, 217	Xylitol	(TMS)_5_	2.117 *	1.312	2.777 *	STD
10.86	45, 73, 103, 129, 147, 217	Adonitol	(TMS)_5_	2.674 *	1.038	2.776 *	STD
10.93	45, 73, 147, 211, 229	Aconitic acid	(TMS)_3_	2.147 *	1.424	3.058 *	Lib
11.27	45, 73, 147, 217, 292	Ribonic acid	(TMS)_5_	2.659 *	1.683 *	4.475 *	Lib
11.68	45, 51, 73, 75, 105, 206	Hippuric acid	TMS	0.656	3.835 *	2.516 *	Lib
12.10	45, 73, 103, 147, 160, 205, 319	Mannose	(TMS)_5_	2.744 *	1.652 *	4.534 *	STD
12.32	45, 73, 103, 117, 147, 205, 217, 319	Mannitol	(TMS)_6_	2.675 *	1.859	4.971 *	STD
13.63	45, 73, 89, 100, 103, 117, 129, 147, 205, 217, 319	D-Allose	(TMS)_5_	2.625 *	1.407	3.695 *	Lib
14.73	73, 103, 147, 217, 269, 357	Pseudo uridine	(TMS)_5_	2.237 *	1.358	3.038 *	Lib
Caffeine Metabolites ^(3)^							
11.82	45, 73, 103, 147, 191, 204, 217	Caffeine	-				Lib
12.22	45, 73, 84, 100, 135, 147, 237, 252 319	Theophylline	TMS				Lib
Not Identified							
5.74	45, 59, 73, 89, 100, 104, 119, 147	N.I.	-	1.746	0.442 *	0.772	
8.81	45, 59, 73, 86, 103, 117, 133, 147, 174, 218	N.I.	-	2.046	2.837 *	5.804 *	
8.83	45, 73, 115, 147	N.I.	-	3.495 *	1.133	3.958 *	
9.30	45, 73, 75, 113, 117, 147	N.I.	-	3.087 *	1.239	3.826 *	
9.74	55, 73, 98, 115, 147, 171, 260	N.I.	-	1.313	3.657 *	4.801 *	
10.70	45, 73, 75, 100, 129, 147, 157, 175	N.I.	-	2.208 *	1.290	2.849 *	
10.89	45, 73, 117, 147, 174	N.I.	-	2.322 *	1.240	2.880 *	
11.75	55, 73, 117, 129, 147, 217, 292	N.I.	-	1.925	2.092 *	4.027 *	
12.57	73, 103, 147, 205, 292	N.I.	-	4.382 *	1.964 *	8.606 *	
12.80	73, 100, 117, 103, 129, 147, 217, 292, 333	N.I. ^(3)^	-				
12.88	45, 73, 75, 83, 100, 117, 147, 241, 344	N.I.	-	1.108	1.442 *	1.597 *	
13.17	45, 73, 83, 100, 142, 267, 282	N.I. ^(3)^	-				
13.47	45, 73, 100, 147, 259, 266, 325, 340	N.I. ^(3)^	-				
13.49	45, 73, 100, 129, 147, 189, 201, 259, 319	N.I.	-	5.822 *	1.128	6.567 *	
13.58	45, 59, 73, 86, 100, 117, 129, 147, 174	N.I.	-	4.131 *	0.659	2.720 *	
14.03	45, 73, 84, 100, 141, 325, 340	N.I. ^(3)^	-				
14.11	45, 73, 100, 147, 383, 398	N.I. ^(3)^	-				

^(1)^ Fold change was calculated by dividing the mean of the peak intensity of each metabolite from each of the two groups; ^(2)^ Ref: Reference, STD: commercial standard, Lib: commercial library; ^(3)^ Detected only in the high fat diet with caffeine (HFDC) group; RT, Retention time; N.I., Not identified; ND, normal diet; HFD, high fat diet; HFDC, high fat diet with caffeine; * *p* value < 0.05, - not detected.

**Table 4:** Discriminated metabolites of the serum identified using UPLC-Q-TOF-MS.

RT (min)	Measured MS (*m/z*)		Tentative Metabolites	HMDB Formula	Error (mDa)	Adduct	Fold Change ^(1)^		
	Positive	Negative					HFD/ND	HFDC/HFD	HFDC/ND
6.18	466.3111	464.2991	Glycocholic acid	C_26_H_43_NO_6_	0.3	[M+H]^+^	0.192 *	1.782	0.342
6.84	431.2766	407.2806	Cholic acid	C_24_H_40_O_5_P	0.0	[M+H]^+^	0.279 *	0.348	0.097 *
8.17	494.3225	538.3101	LysoPC 16:1	C_24_H_50_NO_7_P	0.3	[M+H]^+^	0.684 *	1.035	0.708
9.98	524.3714	568.3626	LysoPC 18:0	C_26_H_54_NO_7_P	1.6	[M+H]^+^	1.279 *	0.887	1.135
Caffeine metabolites ^(2)^									
3.13	181.0727	-	Theobromine	C_7_H_8_N_4_O_2_	0.1	[M+H]^+^			
3.24	197.0735	195.0514	1,7-Dimethyluric acid	C_7_H_8_N_4_O_3_	0.8	[M+H]^+^			
3.37	181.0720	179.0554	Paraxanthine	C_7_H_8_N_4_O_2_	-0.8	[M+H]^+^			
Not-identified									
4.81	321.1302	-	N.I.	-	-	-	0.248 *	3.400	0.843
6.26	355.2635	-	N.I.	-	-	-	0.092 *	0.039	0.004 *
6.95	357.2794	-	N.I.	-	-	-	0.151 *	0.235 *	0.035 *

**^(1)^** Fold change was calculated by dividing the mean of the peak intensity of each metabolite from each of the two groups; ^(2)^ Detected only in the high fat diet with caffeine (HFDC) group; RT, Retention time; N.I., Not identified; LysoPC: lysophosphatidylcholine, ND, normal diet; HFD, high fat diet; HFDC, high fat diet with caffeine; * *p* value < 0.05, - not detected.

**Table 5:** Discriminated metabolites of the serum identified using LTQ-XL-MS.

No.	*m/z* (+)	Adduct	Tentative ID	Fold Change ^(1)^		
				HFD/ND	HFDC/HFD	HFDC/ND
1	468.4	[M+H]^+^	LysoPC 14:0	1.224	0.719 *	0.880
2	508.5	[M+H]^+^	LysoPC P-18:0	1.446 *	0.906	1.310 *
3	520.5	[M+H]^+^	LysoPC 18:2	0.677 *	1.133	0.767 *
4	524.5	[M+H]^+^	LysoPC 18:0	1.596 *	0.971	1.550 *
5	542.5	[M+H]^+^	LysoPC 20:5	0.681 *	1.059	0.721 *
6	546.5	[M+H]^+^	LysoPC 20:3	1.402 *	0.937	1.314 *
7	640.5	[M+NH_4_]^+^	CE 16:1, DG 36:1	0.387 *	1.748 *	0.677
8	642.3	[M+NH_4_]^+^	CE 16:0, DG 36:0	0.624 *	1.660 *	1.035
9	690.2	[M+NH_4_]^+^	CE 20:4	0.784	1.548 *	1.214
10	692.4	[M+NH_4_]^+^	CE 20:3	0.816	1.491 *	1.216
11	694.5	[M+NH_4_]^+^	CE 20:2	0.578 *	1.133	0.654 *
12	764.6	[M+NH_4_]^+^	TG 44:2	1.822 *	0.581 *	1.059
13	780.6	[M+Na]^+^	PC 34:2	0.663 *	1.084	0.719 *
14	784.7	[M+Na]^+^	PC 34:0	0.744 *	1.088	0.809 *
15	794.6	[M+NH_4_]^+^	TG 46:1	1.519 *	1.033	1.569 *
16	810.7	[M+NH_4_]^+^	TG 47:0	1.454 *	1.152	1.675 *
17	822.6	[M+NH_4_]^+^	TG 48:1	1.665 *	0.744	1.239 *
18	838.7	[M+NH_4_]^+^	TG 49:0	1.574 *	0.925	1.456 *
19	842.6	[M+NH_4_]^+^	TG 50:5	0.637 *	1.009	0.642 *
20	844.6	[M+NH_4_]^+^	TG 50:4	0.633 *	0.984	0.622 *
21	850.7	[M+NH_4_]^+^	TG 50:1	2.070 *	0.925	1.915 *
22	852.7	[M+NH_4_]^+^	TG 50:0	2.032 *	0.982	1.995 *
23	860.6	[M+NH_4_]^+^	TG 51:3	0.741 *	0.973	0.721 *
24	870.6	[M+NH_4_]^+^	TG 52:5	0.364 *	0.976	0.355 *
25	872.6	[M+NH_4_]^+^	TG 52:4	0.495 *	1.045	0.517 *
26	876.7	[M+NH_4_]^+^	TG 52:2	2.197 *	1.058	2.324 *
27	878.7	[M+NH_4_]^+^	TG 52:1	2.798 *	1.050	2.939 *
28	880.7	[M+NH_4_]^+^	TG 52:0	1.733 *	1.077	1.866 *
29	886.6	[M+NH_4_]^+^	TG 53:4	0.710 *	1.062	0.753 *
30	892.6	[M+NH_4_]^+^	TG 53:1	0.555 *	0.873	0.485 *
31	896.6	[M+NH_4_]^+^	TG 54:6	0.551 *	0.963	0.531 *
32	898.6	[M+NH_4_]^+^	TG 54:5	0.682 *	1.089	0.742 *
33	904.7	[M+NH_4_]^+^	TG 54:2	1.901 *	1.209	2.299 *
34	906.7	[M+NH_4_]^+^	TG 54:1	1.663 *	1.214	2.019 *
35	916.6	[M+NH_4_]^+^	TG 56:10	0.451 *	0.985	0.444 *
36	918.6	[M+NH_4_]^+^	TG 56:9	0.244 *	1.199	0.293 *
37	920.6	[M+NH_4_]^+^	TG 56:8	0.399 *	1.335	0.532 *
38	922.6	[M+NH_4_]^+^	TG 56:7	0.605 *	1.281	0.775 *
39	928.7	[M+NH_4_]^+^	TG 56:4	1.475 *	1.180	1.741 *
40	942.6	[M+NH_4_]^+^	TG 58:11	0.263 *	1.196	0.315 *
41	944.6	[M+NH_4_]^+^	TG 58:10	0.407 *	1.554 *	0.633 *
42	946.6	[M+NH_4_]^+^	TG 58:9	0.500	1.518 *	0.759 *

**^(1)^** Fold change was calculated by dividing the mean of the peak intensity of each metabolite from each of the two groups; ND, normal diet; HFD, high fat diet; HFDC, high fat diet with caffeine; CE, cholesteryl ester; DG, diacylglycerol; LysoPC, lysophosphatidylcholine; PC, phosphatidylcholine; TG, triglyceride; * *p* value < 0.05.

In the LTQ-XL-MS analysis, the levels of selected CE in our study were decreased. CE is synthesized from cholesterol by acyl-coenzyme A: cholesterol acyltransferase (ACAT), and is degraded by cholesterol ester hydrolase (CEH) to maintain hepatic cholesterol and cholesterol ester homeostasis [42]. In previous research, the CE value increased in the HFD group due to the up-regulation of ACAT [44,45]. However, the level of CEH activity was reported to have increased about 3-fold in the presence of hepatic damage [46]. Thus, the level of CE might be affected by CEH activity, because HFD is a risk factor for hepatic disease [18].

DG is a metabolite repeatedly identified in our study, and consists of two fatty acids produced by phospholipase C. It is also a precursor of TG, a known risk factor for obesity, and is synthesized by acyl CoA: diacylglycerol acyltransferase 1 (DGAT1) [47,48,49]. In this study, the TG content in the serum was observed to increase; however, the DG level was decreased according to the MS analysis data. One possible reason for the difference in DG and TG levels might be due to the activation of DGAT1. When TG accumulates such as in obesity due to a HFD, DGAT1 increases the TG level to conjugate excess of fatty acids in the liver. Zhao *et al.* [50] reported that the level of TG and DGAT1 gene expression increased in the fat Banna Mini-pig Inbred Line (BMIL) compared with the lean BMIL. Thus, the increased TG level seems to be due to DGAT1 expression in the HFD group. However, not all of the 29 TGs showed a significant difference in this study; 13 TGs were increased, while the other 16 TGs were decreased in the HFD group. This suggests that the TGs were also changed by HFD consumption, and each TG level might change in a different pattern. These changes are yet to be elucidated.

Bile acids, which play an important role in cholesterol synthesis, are synthesized from cholesterol by cholesterol 7-α-hydroxylase (CYP7A1) and secreted into the intestine and blood. Because the enzyme CYP7A1 increases in a HFD, the serum bile acid level is increased [51]. Previous reports suggested that inflammatory factors such as tumor necrosis factor-alpha (TNF-α) and interleukins (ILs) that are induced by a HFD decrease CYP7A1 mRNA levels and activity [52,53]. Thus, inflammation induced by HFD ultimately decreases the content of bile acids such as cholic acid and glycocholic acid, which was observed in our study. 

In the HFD, the body’s lipid metabolism becomes abnormal and dysfunctional due to the up- or down-regulated expression of various genes [42,54] and nutrients. For example, fatty acids activate pro-inflammatory gene transcription [55]. Thus, we inferred that the level of lipids such as lysoPC, PCs, CEs, DGs, TGs, and bile acid in our study was affected by perturbations of lipid metabolism induced by the HFD, but further studies are required to elucidate the relationship between the HFD and lipid metabolites related mechanism. 

A number of the serum metabolites altered by consuming caffeine were lower than those altered by consuming the HFD. Among the metabolites that were significantly different in the HFD group, lysoPC 14:0 and TG 44:2 were significantly decreased, while CE 16:1, CE16:0, CE 20:4, CE 20:3, DG 36:1, DG 36:0, TG 58:10, and TG 58:9 were increased in HFDC group. The latter metabolites were decreased in the HFD group. Based on these results, we inferred that those lipid metabolites were potential biomarkers of HFD as well as caffeine consumption. Therefore, our study indicates that caffeine has an anti-obesity effect, and it was referred to the changes of several metabolites associated with the lipid metabolism. Further study related to enzyme activity and gene expression has to be performed in order to achieve a more detailed picture of lipid metabolism in a HFD with or without caffeine consumption.

### 2.4. Analysis of Caffeine Metabolites in Urine and Serum

Among the metabolites that were significantly different between each group, some were only detected in the urine and serum of the HFDC group by UPLC-Q-TOF-MS and GC-TOF-MS. These metabolites were identified as caffeine-related metabolites and presented in Table 2, Table 3, and Figure S2. Twelve caffeine metabolites, including 5-acetylamino-6-amino-3-methyluracil (AAMU), 1-methyluric acid, 1 or 3 or 7-methylxanthine, 3,7 or 1,3 or 1,7-dimethyluric acid, 1,3,7-trimethyluric acid, theobromine, paraxanthine, and caffeine were observed in the urine of the HFDC group. In the serum, theobromine, 1,7-dimethyluric acid, and paraxanthine were observed and identified in the HFDC group. Caubet *et al.* reported that ^13^C-caffeine metabolites can be identified in urine by LC-MS [56]. We detected major caffeine metabolites such as paraxanthine and theobromine in the urine and serum, meaning that caffeine metabolism did occur in the HFDC group.

## 3. Experimental Section 

### 3.1. Reagents

HPLC-grade water, methanol, chloroform, and acetonitrile were purchased from Fisher Scientific (Pittsburgh, PA, USA). Pyridine, methoxyamine hydrochloride, *N*-methyl-*N*-(trimethylsilyl) trifluoroacetamide (MSTFA), and urease were purchased from Sigma Aldrich (St. Louis, MO, USA).

### 3.2. Animals and Diet

Four-week-old Sprague-Dawley rats (*n* = 29) were purchased from Daehan bio-link (Chungbuk, Korea). All rats were housed with two rats per cage in a climate-controlled room (temperature: 22 ± 2 °C; relative humidity: 55% ± 5%; 24 h air circulation via forced ventilation) on a 12 h light/12 h dark photocycle. All experiments were performed according to the policies and guidelines of the Institutional Animal Care and Use Committee (IACUC) at Kyung Hee University, Yongin, Korea (approval number: KHUASP(SE)14-19). During the one-week adaptation period, rats were allowed free access to a normal diet and water. After 1 week, 10 rats were fed a ND for 12 weeks, and 19 rats were fed a HFD to induce obesity. The ND group was fed Harlan 2018S (3.1 kcal/g, 44.2% carbohydrate, 18.6% protein, and 6.2% fat; Teklad Diets, Madison WI, USA), and the HFD group was fed 60 kcal% fat (lard; 5.1 kcal/g, 21.3% carbohydrate, 18.4% protein, and 60.3% fat; TD.06414, Harlan) *ad libitum*. After 12 weeks, the 19 rats were randomized into two groups to investigate the effect of caffeine consumption on obesity for 9 weeks: (1) HFD; HFD + ultra-pure water (*n* = 9), and (2) HFDC; HFD + 0.1% caffeine solution (*n* = 10). During the 9-week period, food intake was measured daily, and body weight was measured twice a week. The caffeine or water solution was changed every other day.

### 3.3. Urine, Serum, Organ, and Adipose Tissue Sample Preparation

The rats were transferred to individual metabolic cages for urine collection for a 12 h fasting period. During this time, the rats had free access to water. Urine was collected and stored at −70 °C until analysis. At the end of the fasting period, rats were anesthetized with diethyl ether. Blood was collected by cardiac puncture using heparin-treated syringes and centrifuged at 2000 rpm for 15 min at 4 °C. After centrifugation, the supernatant was separated and stored at −70 °C until analyzed. The adipose tissue and abdominal adipose tissue were then immediately frozen in liquid nitrogen and stored at −70 °C until analysis. Abdominal adipose tissue was fixed in 10% formalin and embedded in paraffin, followed by deparaffinization and rehydration. The paraffin blocks were stained with hematoxylin and eosin (H&E). Abdominal adipose tissue sections were viewed with a fluorescence microscope (Axiovert s100, Carl-Zeiss, Göttingen, Germany).

### 3.4. Analyses of Serum, Urine, Liver, and Adipose Samples

#### 3.4.1. Serum

Serum TC, HDL-C, and TG levels were measured enzymatically using a commercial kit (Asan Pharmaceutical Co., Seoul, Korea). LDL-C levels were determined using the following formula: LDL-C = TC − (1/5 × TG + HDL-C). Fatty acid concentration was determined using a NEFA-HR II (Wako Pure Chemical Industries, Osaka, Japan).

#### 3.4.2. Liver Lipid

Hepatic lipid was extracted using a method described by Folch *et al.* [57]. The liver (0.5 g) was homogenized in 1.5 mL of 0.9% saline. Afterwards, 7.5 mL of the following mixture of chloroform–methanol (1:2, *v/v*; 7.5 mL) was added to the homogenate. The hepatic TG and TC were measured by enzymatic colorimetric methods using commercial kits (Asan Pharmaceutical, Seoul, Korea).

#### 3.4.3. Abdominal Adipose Tissue Enzyme Activity

The GPDH activity was quantified using a colorimetric assay kit (BioVision Research Products, Mountain View, CA, USA). Briefly, abdominal tissue was homogenized with 200 µL of ice-cold GPDH assay buffer for 10 min on ice and then centrifuged at 12,000 rpm for 5 min. The supernatant was collected. One unit of specific enzyme activity corresponded to the oxidation of 1 nmol NADH/min/mg of protein. The LPL activity was measured using a fluorometric assay (Cell Biolabs, Inc., San Diago CA, USA). Abdominal tissues were minced and homogenized in 1 mL of cold 20 mM Tris at pH 7.5 and 150 mM NaCl, and then centrifuged at 12,000 rpm for 10 min at 4 °C. Enzyme activity was expressed as nmol of fatty acids released/min/mg of protein. Protein was measured using the Bradford reagent from Sigma-Aldrich Co.

### 3.5. The Preparation of Urine and Serum Samples for MS Analysis

Urine samples for UPLC-Q-TOF-MS and GC-TOF-MS analysis were prepared using the protocols described by Want [58] and Chan [59], respectively. Prior to UPLC-Q-TOF-MS analysis, particulates were removed from the urine using a 0.2-μm polytetrafluoroethylene (PTFE) filter. Following filtration, 150 µL of urine and 150 µL of water were mixed and re-filtered. For GC-TOF-MS analysis, 150 µL of urine was added to 20 µL of urease suspension (100 U of urease) and incubated at 37 °C for 30 min to remove the excess urea. The enzymatic reaction was stopped with the addition of 600 µL of methanol and slight vortexing. Samples were then centrifuged at 10,000 rpm for 10 min. A total of 200 µL of supernatant was evaporated to dryness and then derivatized through the subsequent addition of pyridine with methoxyamine (20 mg/mL, *v*/*v*) and MSTFA at 60 °C for 2 and 1 h, respectively. 

Serum was extracted before UPLC-Q-TOF-MS analysis. A total of 900 µL of ice-cold methanol was added to the 300 µL of serum sample, which was then homogenized for 5 min using a mixer mill (Retsch GmbH & Co, KG, Haan, Germany). The mixture was kept at −20 °C for 1 h and then centrifuged at 12,000 rpm for 10 min at 4 °C. The resulting supernatant was filtered through a syringe filter (0.2 µm) and transferred to an Eppendorf tube. The remaining pellet was extracted again using 900 µL of chloroform. The chloroform layers were combined with the methanol extracts and evaporated with a speed-vacuum machine. The dried sample was dissolved in methanol and filtered through a 0.2-µm PTFE filter for UPLC-Q-TOF-MS analysis. 

To analyze serum lipids by LTQ-XL-MS, sera were extracted using the Matyash method with modifications [60,61]. In brief, 225 µL of ice-cold methanol was added to a 10 µL of serum sample, and the mixture vortexed for 10 s. Subsequently, 750 µL of ice-cold methyl-*tert*-butyl ether (MTBE) was added, and phase separation was induced through the addition of 187.5 µL of distilled water. Following centrifugation at 10,000 rpm at 4 °C, the supernatant was collected and dried completely. Dried lipid extracts were reconstituted with 100 µL of chloroform–methanol (1:9, *v/v*) and diluted 10-fold with chloroform–methanol (1:9, *v/v*) containing 7.5 mM ammonium acetate.

### 3.6. Instrumental Analysis

#### 3.6.1. UPLC-Q-TOF-MS Analysis

The UPLC-Q-TOF-MS analysis of the urine and serum samples was performed using a Waters Micromass Q-TOF Premier with UPLC Acquity System^TM^ (Waters, Milford, MA, USA). Chromatographic analysis was performed using a Waters Acquity Tunable UV (TUV), sample manager (autosampler), and binary solvent manager (pump), with the Acquity UPLC BEH C_18_ column (100 × 2.1 mm, 1.7 μm, Waters). The binary gradient system of acetonitrile (0.1% acetic acid) and water (0.1% acetic acid) was initiated with 0% acetonitrile for 0.3 min. The gradient was gradually increased to 30% acetonitrile over 3 min to 40% acetonitrile for 1 min, and finally to 100% acetonitrile for 8 min. The mobile phase was maintained at 100% acetonitrile for 2 min, followed by 0% acetonitrile for 2 min. The injection volume was 5 μL, and the flow rate was maintained at 0.3 mL/min. The mass spectrometry system was set up with the following parameters: ion source temperature, 200 °C; cone gas flow, 50 L/h; desolvation gas flow, 600 L/h; capillary voltage, 2.8 kV; and cone voltage, 35 V. Negative and positive modes were analyzed within a 100–1000 range of *m/z*.

#### 3.6.2. GC-TOF-MS Analysis

The GC-TOF-MS analyses were performed using Leco TOF Pegasus III mass spectrometry (Leco, St. Joseph, MI, USA) operating in electron ionization (EI) mode (70 eV) with an Agilent 7890A GC system (Palo Alto, CA, USA). The column was a DB-5MS (30 m length × 0.25 mm i.d. × 0.25 μm film thickness, J & W Scientific, Folsom, CA, USA). Helium was used as the carrier gas and had a constant flow of 1.5 mL/min. A total of 1 μL of the derivatized sample was injected in a split mode (10:1). The oven temperature was sustained at 75 °C for 2 min and then increased to 300 °C at a rate of 15 °C/min. The temperature was then maintained at 300 °C for 3 min. The injector and ion source temperatures were 250 and 230 °C, respectively. The acquisition rate was 20 scans/s, with a mass scan range of *m/z* 45–1000. 

#### 3.6.3. LTQ-XL-MS Lipid Analysis

Serum lipid profiling was performed by LTQ XL mass spectrometry (Thermo Fischer Scientific, West Palm Beach, FL, USA) equipped with a robotic nanoflow ion source, TriVersa Nanomate (Advion Biosciences, Ithaca, NY, USA), and used nanoelectrospray chips with a spraying nozzle diameter of 5.5 μm. The ion source was controlled by Chipsoft 8.3.1 software (Advion Biosciences). The ionization voltage was −1.45 kV in negative mode; backpressure was set at 0.4 psi. The temperature of the ion transfer capillary was 200 °C, and the tube voltage was −100 V. A total of 10 μL of reconstituted lipid extracts were loaded onto TriVersa Nanomate ion source of 96-well plates sealed with aluminum foil. Each sample was analyzed for 2 min. The data collection method performed a full scan (scan range: *m/z* 400–1000) and a data dependent MS/MS scan of the most abundant ions. All spectra were recorded using the Thermo Xcalibur software (version 2.1, Thermo Fisher Scientific, San Jose, CA, USA).

### 3.7. Data Processing and Statistical Analysis

The UPLC-Q-TOF-MS and GC-TOF-MS raw data were converted to netCDF format using databridge software (Waters) and ChromaTOF software (LECO), respectively. Following the conversion, the MS data were processed using the Metalign software package (version 200410, Wageningen, Netherlands, http://www.metalign.nl). The resulting data were then exported to Excel (Microsoft, Redmond, WA, USA), and statistical analysis was performed using SIMCA-P + 12.0 software (Umetrics, Umea, Sweden). Lipid MS data obtained from LTQ XL ion trap mass spectrometry were aligned using MATLAB software (version 8.0, MathWorks, Natick, MA, USA), and raw files were used to obtain a data matrix containing *m/z* values and peak intensities for multivariate analysis. The datasets were auto-scaled (unit variance scaling), log-transformed, and mean-centered in a column-wise fashion. PLS-DA was used to compare the three groups and to identify the major metabolites in each group. Variables with VIP values greater than 0.7 and *p* values less than 0.05 were considered to be significant. The significantly different metabolites were matched to the references and the human metabolome database [62]. In addition, MS/MS spectra were analyzed for the identification of lipid species using the LIPID MAPS Lipidomics Gateway [63], LipidBlast [64], and an in-house library. All histological data were presented as mean ± SD. Statistical calculations were performed with SPSS (Statistical Package for the Social Science; version 21.0 for Windows, Chicago, IL, USA). Experimental groups were compared using one-way ANOVA and Tukey’s multiple range tests, with *p* values < 0.05 considered significant.

## 4. Conclusions

Our study investigated the changes in clinical factors and biofluid metabolites that resulted from a HFD or HFDC-fed rat. In the clinical data of serum and liver, the factors affected by the HFD were partially reversed by caffeine consumption. Various metabolites such as glucuronide-conjugated compounds, lysoPCs, PCs, CEs, DGs, TGs, bile acids, sugars, pseudouridine, creatinine, taurine, and hippuric acid in the urine and serum were significantly altered by the HFD. Among them, the alteration of some glucuronide-conjugated compounds, lysoPCs, CEs, DGs, TGs, taurine, and hippuric acid were moderated by caffeine consumption. In this study, we demonstrated the effect caffeine has on HFD-induced obesity. Furthermore, the MS-based metabolomics technique gave detailed information about changes in each of the metabolites. We also suggested potential biomarker candidates in the urine and serum that were affected by the HFD and caffeine consumption.

## Supplementary Materials

Supplementary materials can be accessed at: http://www.mdpi.com/1420-3049/20/02/3107/s1.

## References

[1] Malnick S.D.H., Malnick D.H., Knobler H. (2006). The medical complications of obesity. QJM.

[2] Mokdad A.H., Bowman B.A., Ford E.S., Vinicor F., Marks J.S., Koplan J.P. (2001). The continuing epidemics of obesity and diabetes in the United States. JAMA.

[3] Park Y.R., Cho Y.G., Kang J.H., Park H.A., Kim K.W., Hur Y.I., Seo J.S., Park N.Y. (2014). Comparison of Obesity and Overweight Prevalence Among Korean Adults According to Community Health Survey and Korea National Health and Nutrition Examination Survey. Korean J. Obes..

[4] Bae N.K., Kwon I.S., Cho Y.C. (2009). Ten Year Change of Body Mass Index in Korean: 1997–2007. Korean J. Obes..

[5] Martínez I., Perdicaro D.J., Brown A.W., Hammons S., Carden T.J., Carr T.P., Eskridge K.M., Walter J. (2013). Diet-induced alterations of host cholesterol metabolism are likely to affect the gut microbiota composition in hamsters. Appl. Environ. Microbiol..

[6] DeAngelis R.A., Markiewski M.M., Taub R., Lambris J.D. (2005). A high-fat diet impairs liver regeneration in C57BL/6 mice through overexpression of the NF-kappaB inhibitor, IkappaBalpha. Hepatology.

[7] Backhed F., Manchester J.K., Semenkovich C.F., Gordon J.I. (2007). Mechanisms underlying the resistance to diet-induced obesity in germ-free mice. Proc. Natl. Acad. Sci. USA.

[8] Kim H.J., Kim J., Noh S., Hur H.J., Sung M.J., Hwang J.T., Park J.H., Yang H.J., Kim M.S., Kwon D.Y. (2011). Metabolomic analysis of livers and serum from high-fat diet induced obese mice. J. Proteome Res..

[9] Bressanelloa D., Libertoa E., Collinoa M., Reichenbach S.E., Benetti E., Chiazza F., Cordero C. (2014). Urinary metabolic fingerprinting of mice with diet-induced metabolic derangements by parallel dual secondary column-dual detection two-dimensional comprehensive gas chromatography. J. Chromatogr. A.

[10] Connor S.C., Hansen M.K., Corner A., Smith R.F., Ryan T.E. (2010). Integration of metabolomics and transcriptomics data to aid biomarker discovery in type 2 diabetes. Mol. BioSyst..

[11] Shah S.H., Kraus W.E., Newgard C.B. (2012). Metabolomic profiling for the identification of novel biomarkers and mechanisms related to common cardiovascular diseases: Form and function. Circulation.

[12] Denkert C., Bucher E., Hilvo M., Salek R., Orešič M., Griffin J., Brockmöller S., Klauschen F., Loibl S., Barupal D.K. (2012). Metabolomics of human breast cancer: new approaches for tumor typing and biomarker discovery. Genome Med..

[13] Kaur P., Sheikh K., Alexander K., Kirilyuk K., Singh R., Ressom H.W., Cheema A.K. (2012). Metabolomic profiling for biomarker discovery in pancreatic cancer. Int. J. Mass Spectrom..

[14] Tomita R., Todoroki K., Machida K., Nishida S., Maruoka H., Yoshida H., Fujioka T., Nakashima M., Yamaguchi M., Nohta H. (2014). Assessment of the efficacy of anticancer drugs by amino acid metabolomics using fluorescence derivatization-HPLC. Anal. Sci..

[15] Yang Z., Marotta F. (2012). Pharmacometabolomics in drug discovery & development: Applications and challenges. Metabolomics.

[16] Mohanpuria P., Kumar V., Yadav S.K. (2010). Tea caffeine: metabolism, functions, and reduction strategies. Food Sci..

[17] Paradkar M.M., Irudayaraj J. (2002). Rapid determination of caffeine content in soft drinks using FTIR–ATR spectroscopy. Food Chem..

[18] Panchal S.K., Wong W.Y., Kauter K., Ward L.C., Brown L. (2012). Caffeine attenuates metabolic syndrome in diet-induced obese rats. Nutrition.

[19] Vercambre M.N., Berr C., Ritchie K., Kang J.H. (2013). Caffeine and cognitive decline in elderly women at high vascular risk. J. Alzheimers Dis..

[20] Kobayahi-Hattori K., Mogi A., Matsumoto Y., Takita T. (2005). Effect of caffeine on the body fat and lipid metabolism of rats fed on a high-fat diet. Biosci. Biotechnol. Biochem..

[21] Sugiura C., Nishimatsu S., Moriyama T., Ozasa S., Kawada T., Sayama K. (2012). Catechins and caffeine inhibit fat accumulation in mice through the improvement of hepatic lipid metabolism. J. Obes..

[22] Zhang X.J., Chinkes D.L., Aarsland A., Herndon D.N., Wolfe R.R. (2008). Lipid metabolism in diet-induced obese rabbits is similar to that of obese humans. J. Nutr..

[23] Kim J.Y., Park J.Y., Kim O.Y., Ham B.M., Kim H.J., Kwon D.Y., Jang Y., Lee J.H. (2010). Metabolic profiling of plasma in overweight/obese and lean men using ultra performance liquid chromatography and Q-TOF mass spectrometry (UPLC-Q-TOF MS). J. Proteome Res..

[24] Kusunoki M., Tsutsumi K., Sato D., Nakamura T. (2012). Lipoprotein lipase and obesity. Health.

[25] Xu S.P., Mao X.Y., Ren F.Z., Che H.L. (2011). Attenuating effect of casein glycomacropeptide on proliferation, differentiation, and lipid accumulation of *in vitro* Sprague-Dawley rat preadipocytes. J. Dairy Sci..

[26] Wang H., Eckel R.H. (2009). Lipoprotein lipase: From gene to obesity. Am. J. Physiol. Endocrinol. Metab..

[27] Moy G.A., McNay E.C. (2013). Caffeine prevents weight gain and cognitive impairment caused by a high-fat diet while elevating hippocampal BDNF. Physiol. Behav..

[28] Sinha R.A., Farah B.L., Singh B.K., Siddique M.M., Li Y., Wu Y., Ilkayeva O.R., Gooding J., Ching J., Zhou J. (2014). Caffeine stimulates hepatic lipid metabolism by the autophagy-lysosomal pathway in mice. Hepatology.

[29] King C.D., Rios G.R., Green M.D., Tephly T.R. (2000). UDP-glucuronosyltransferases. Curr. Drug Metab..

[30] Cani P.D., Bibiloni R., Knauf C., Waget A., Neyrinck A.M., Delzenne N.M., Burcelin R. (2008). Changes in gut microbiota control metabolic endotoxemia-induced inflammation in high-fat diet–induced obesity and diabetes in mice figure legends. Diabetes.

[31] Erridge C., Attina T., Spickett C. M., Webb D.J. (2007). A high-fat meal induces low-grade endotoxemia: Evidence of a novel mechanism of postprandial inflammation. Am. J. Clin. Nutr..

[32] Kim K.A., Gu W., Lee I.A., Joh E.H., Kim D.H. (2012). High fat diet-induced gut microbiota exacerbates inflammation and obesity in mice via the TLR4 signaling pathway. PLoS One.

[33] Zhang L., Chu X., Wang H., Xie H., Guo C., Cao L., Zhou X., Wang G., Hao H. (2013). Dysregulations of UDP-glucuronosyltransferases in rats with valproic acid and high fat diet induced fatty liver. Eur. J. Pharmacol..

[34] Congiu M., Mashford M.L., Slavin J.L., Desmond P.V. (2002). UDP glucuronosyltransferase mRNA levels in human liver disease. Drug Metab. Dispos..

[35] Elwakkad A., Al-azhary D., Mohamed S. (2011). The enhancement of the anti-inflammatory effect of caffeine on green tea extract and EGCG on obese rats. J. Am. Sci..

[36] An Y., Xu W., Li H., Lei H., Zhang L., Hao F., Duan Y., Yan X., Zhao Y., Wu J. (2013). High-fat diet induces dynamic metabolic alterations in multiple biological matrices of rats. J. Proteome Res..

[37] Wu Q., Zhang H., Dong X., Chen X.F., Zhu Z.Y., Hong Z.T., Chai Y.F. (2014). UPLC-Q-TOF/MS based metabolomic profiling of serum and urine of hyperlipidemic rats induced by high fat diet. J. Pharm. Anal..

[38] Jung U.J., Choi M.S. (2014). Obesity and Its Metabolic Complications: The Role of Adipokines and the Relationship between Obesity, Inflammation, Insulin Resistance, Dyslipidemia and Nonalcoholic Fatty Liver Disease. Int. J. Mol. Sci..

[39] Tamura S., Fujioka H., Nakano T., Amuro Y., Hada T., Nakao N., Higashino K. (1988). Urinary pseudouridine as a biochemical marker in the diagnosis and monitoring of primary hepatocellular carcinoma. Am. J. Gastroenterol..

[40] Sun Y.J., Wang H.P., Liang Y.J., Liang Y.J., Yang L., Wu Y.J. (2012). An NMR-based metabonomic investigation of the subacute effects of melamine in rats. J. Proteome Res..

[41] Abbott M.J., Tang T., Sul H.S. (2010). The Role of Phospholipase A2-derived Mediators in Obesity. Drug Discov. Today Dis. Mech..

[42] Yaligar J., Gopalan V., Kiat O.W., Sugii S., Shui G., Lam B.D., Henry C.J., Wenk M.R., Tai E.S., Velan S.S. (2014). Evaluation of dietary effects on hepatic lipids in high fat and placebo diet fed rats by *in vivo* MRS and LC-MS techniques. PLoS One.

[43] Kim H.Y., Kim M., Park H.M., Kim J., Kim E.J., Lee C.H., Yoon-Park J.H. (2014). Lysophospholipid profile in serum and liver by high-fat diet and tumor induction in obesity-resistant BALB/c mice. Nutrition.

[44] Eisinger K., Liebisch G., Schmitz G., Aslanidis C., Krautbauer S., Buechler C. (2014). Lipidomic Analysis of Serum from High Fat Diet Induced Obese Mice. Int. J. Mol. Sci..

[45] Borg M.L., Omran S.F., Weir J., Meikle P.J., Watt M.J. (2012). Consumption of a high-fat diet, but not regular endurance exercise training, regulates hypothalamic lipid accumulation in mice. J. Physiol..

[46] Simon J.B., Poon R.W. (1978). Hepatic cholesterol ester hydrolase in human liver disease. Gastroenterology.

[47] Murase T., Mizuno T., Omachi T., Onizawa K., Komine Y., Kondo H., Hase T., Tokimitsu I. (2001). Dietary diacylglycerol suppresses high fat and high sucrose diet-induced body fat accumulation in C57BL/6J mice. J. Lipid Res..

[48] Hokanson J.E., Austin M.A. (1996). Plasma triglyceride level is a risk factor for cardiovascular disease independent of high-density lipoprotein cholesterol level: A meta-analysis of population-based prospective studies. J. Cardiovasc. Risk.

[49] Cases S., Smith S.J., Zheng Y.W., Myers H.M., Lear S.R., Sande E., Novak S., Collins C., Welch C.B., Lusis A.J. (1998). Identification of a gene encoding an acyl CoA:diacylglycerol acyltransferase, a key enzyme in triacylglycerol synthesis. Proc. Natl. Acad. Sci. USA.

[50] Zhao S.M., Li W.Z., Pan H.B., Huang Y., Yang M.H., Wei H.J., Gao S.Z. (2012). Expression levels of candidate genes for intramuscular fat deposition in two Banna mini-pig inbred lines divergently selected for fatness traits. Genet. Mol. Biol..

[51] Suzuki Y., Kaneko R., Nomura M., Naito H., Kitamori K., Nakajima T., Ogawa T., Hattori H., Seno H., Ishii A. (2013). Simple and rapid quantitation of 21 bile acids in rat serum and liver by UPLC-MS-MS: Effect of high fat diet on glycine conjugates of rat bile acids. Nagoya J. Med. Sci..

[52] Cortez M., Carmo L.S., Rogero M.M., Borelli P., Fock R.A. (2013). A high-fat diet increases IL-1, IL-6, and TNF-α production by increasing NF-κB and attenuating PPAR-γ expression in bone marrow mesenchymal stem cells. Inflammation.

[53] Feingold K.R., Spady D.K., Pollock A.S., Moser A.H., Grunfeld C. (1996). Endotoxin, TNF, and IL-1 decrease cholesterol 7 α-hydroxylase mRNA levels and activity. J. Lipid Res..

[54] Lópeza I.P., Milagrob F.I., MartÍb A., Moreno-Aliagab M.J., MartÍnezb J.A., de Miguel C. (2004). Gene expression changes in rat white adipose tissue after a high-fat diet determined by differential display. Biochem. Biophys. Res. Commun..

[55] Caricilli A.M., Nascimento P.H., Pauli J.R., Tsukumo D.M., Velloso L.A., Carvalheira J.B., Saad M.J. (2008). Inhibition of toll-like receptor 2 expression improves insulin sensitivity and signaling in muscle and white adipose tissue of mice fed a high-fat diet. J. Endocrinol..

[56] Caubet M.S., Comteb B., Brazier J.L. (2004). Determination of urinary 13C-caffeine metabolites by liquid chromatography–mass spectrometry: the use of metabolic ratios to assess CYP1A2 activity. J. Pharm. Biomed. Anal..

[57] Folch J., Lees M., Sloane-Stanley G.H. (1957). A simple method for the isolation and purification of total lipides from animal tissues. J. Biol. Chem..

[58] Want E.J., Wilson I.D., Gika H., Theodoridis G., Plumb R.S., Shockcor J., Holmes E., Nicholson J.K. (2010). Global metabolic profiling procedures for urine using UPLC-MS. Nat. Protoc..

[59] Chan E.C., Pasikanti K.K., Nicholson J.K. (2011). Global urinary metabolic profiling procedures using gas chromatography-mass spectrometry. Nat. Protoc..

[60] Matyash V., Liebisch G., Kurzchalia T.V., Shevchenko A., Schwudke D. (2008). Lipid extraction by methyl-tert-butyl ether for high-throughput lipidomics. J. Lipid Res..

[61] Lee D.Y., Kind K., Yoon Y.R., Fiehn O., Liu K.H. (2014). Comparative evaluation of extraction methods for simultaneous mass-spectrometric analysis of complex lipids and primary metabolites from human blood plasma. Anal. Bioanal. Chem..

[62] Wishart D.S., Jewison T., Guo A.C., Wilson M., Knox C., Liu Y., Djoumbou Y., Mandal R., Aziat F., Dong E. (2013). HMDB 3.0—The Human Metabolome Database in 2013. Nucleic Acids Res..

[63] Fahy E., Sud M., Cotter D., Subramaniam S. (2007). LIPID MAPS online tools for lipid research. Nucleic Acids Res..

[64] Kind T., Liu K.H., Lee D.Y., DeFelice B., Meissen J.K., Fiehn O. (2013). LipidBlast in silico tandem mass spectrometry database for lipid identification. Nat. Methods.

