# Supplementary material for: Urine and Serum Metabolite Profiling of Rats Fed a High-Fat Diet and the Anti-Obesity Effects of Caffeine Consumption
Source: Molecules. 2015 Feb 13;20(2):3107–28. doi: 10.3390/molecules20023107 (PMC6272342; doi:10.3390/molecules20023107)
Supplement: Supplementary file 1 [file molecules-20-03107-s001.pdf]

Supplementary Information

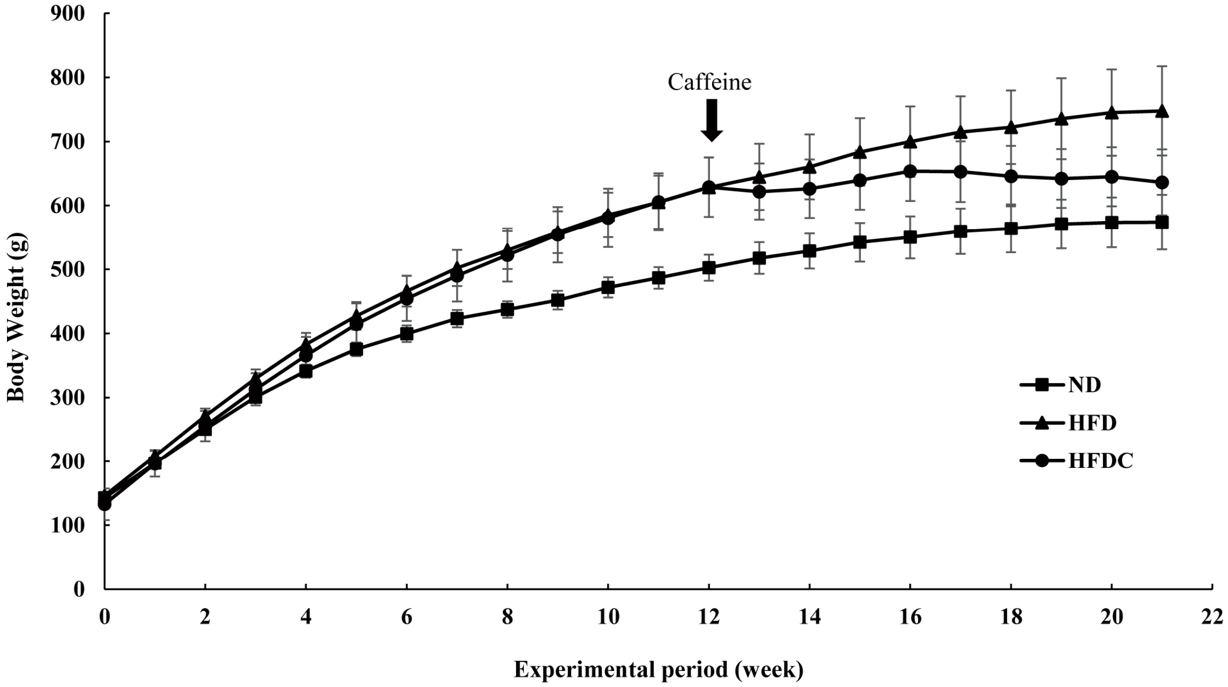

Figure S1. Effects of caffeine on body weight in rats fed a high-fat diet.

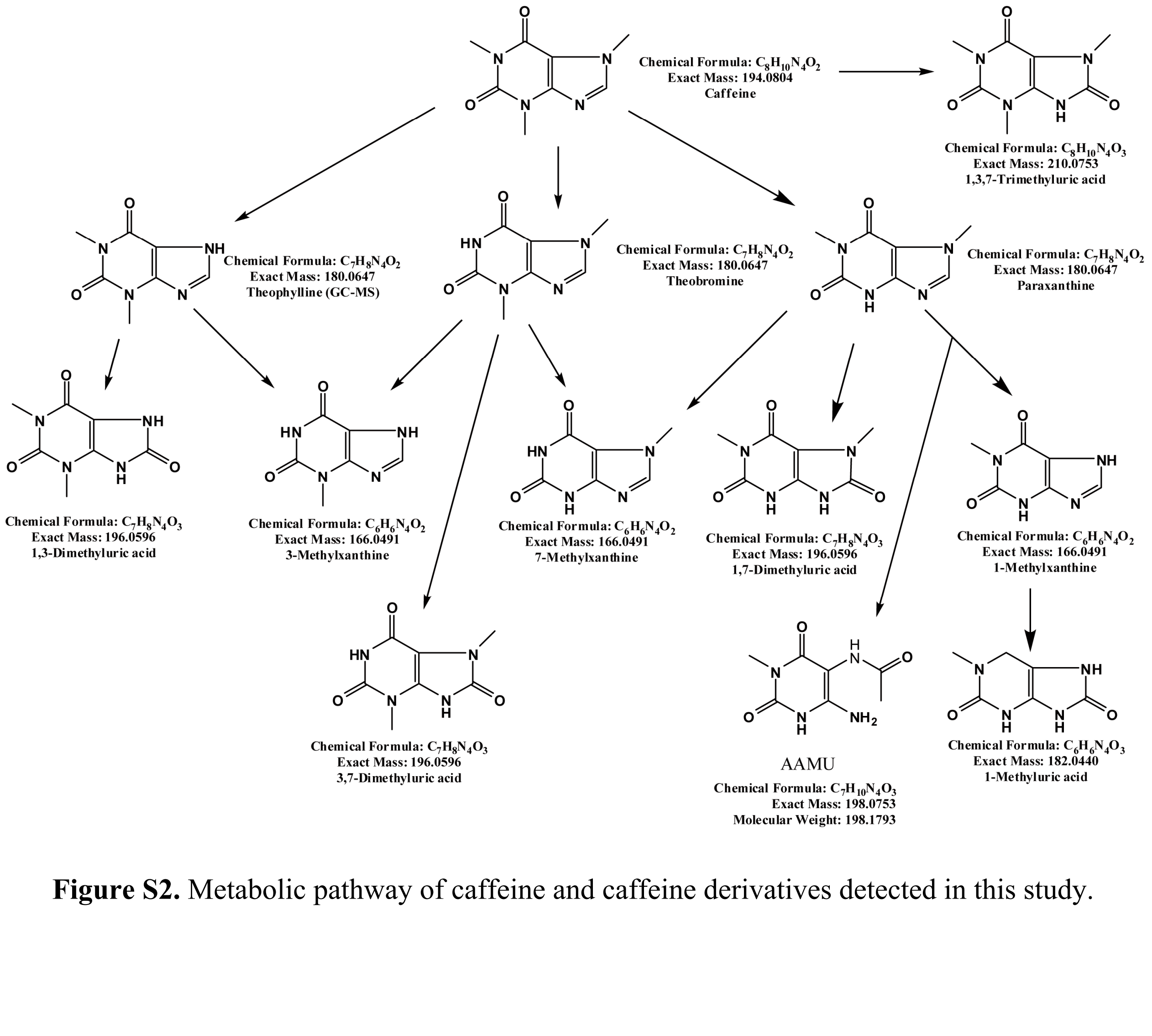

Figure S2. Metabolic pathway of caffeine and caffeine derivatives detected in this study.
